# The Contribution of Complementary and Alternative Medicine to Reduce Antibiotic Use: A Narrative Review of Health Concepts, Prevention, and Treatment Strategies

**DOI:** 10.1155/2019/5365608

**Published:** 2019-02-03

**Authors:** Erik W. Baars, Eefje Belt-van Zoen, Thomas Breitkreuz, David Martin, Harald Matthes, Tido von Schoen-Angerer, Georg Soldner, Jan Vagedes, Herman van Wietmarschen, Olga Patijn, Merlin Willcox, Paschen von Flotow, Michael Teut, Klaus von Ammon, Madan Thangavelu, Ursula Wolf, Josef Hummelsberger, Ton Nicolai, Philippe Hartemann, Henrik Szőke, Michael McIntyre, Esther T. van der Werf, Roman Huber

**Affiliations:** ^1^Louis Bolk Institute, Kosterijland 3-5, 3981 AJ Bunnik, Netherlands; ^2^University of Applied Sciences Leiden, Faculty of Healthcare, Zernikedreef 11, 2333 CK Leiden, Netherlands; ^3^Filderklinik, Im Haberschlai 7, 70794 Filderstadt, Germany; ^4^University of Witten/Herdecke, Alfred-Herrhausen-Straße 50, 58448 Witten, Germany; ^5^Charité Universitätsmedizin Berlin, Institute for Social Medicine, Epidemiology and Health Economics, Luisenstr. 57, 10117 Berlin, Germany; ^6^Department of Pediatrics, Fribourg Hospital HFR, Fribourg, Switzerland; ^7^Medical section of the Goetheanum, Rüttiweg 45 4143 Dornach, Switzerland; ^8^ARCIM institute, Im Haberschlai 7, 70794 Filderstadt, Germany; ^9^University of Southampton, University Road, Southampton SO17 1BJ, UK; ^10^Sustainable Business Institute, Zehnthofstr. 1, 65375 Oestrich-Winkel, Germany; ^11^University of Bern, Freiburgstrasse 46, 3010 Bern, Switzerland; ^12^European Ayurveda Association e.V., In den Forstwiesen 27, D- 56745 Bell, Germany; ^13^Technical University Munich, Georg-Brauchle-Ring 62, 80807 Munich, Germany; ^14^Eurocam, Rue du Trône 194, 1050 Brussels, Belgium; ^15^University of Lorraine, School of Medicine, 7 avenue de la Forêt de Haye, 54500 Vandoeuvre-Nancy, France; ^16^University of Pécs, 7622 Pécs, Vasvári Pál str. 4., Hungary; ^17^Midsummer Clinic, Church Westcote, Chipping Norton, Oxon, Ox7 6SF, UK; ^18^Taylor's University, School of Medicine, 1, Jalan Taylor's, 47500 Subang Jaya, Selangor D.E., Malaysia; ^19^University of Bristol, Bristol Medical School, Canynge Hall, 39 Whatley Road, Bristol BS8 2PS, UK; ^20^University of Freiburg, Faculty of Medicine, Breisacher Str. 115b, 79106 Freiburg, Germany

## Abstract

**Aim:**

The aim of this narrative review was to explore the potential contributions of CAM to reduce antibiotic use.

**Methods:**

We searched PubMed, Embase, and Cochrane Database of Systematic Reviews with a specific, limited set of search terms and collected input from a group of expert CAM researchers to answer the question: What is known about the contribution of CAM health and health promotion concepts, infection prevention, and infection treatment strategies to reduce antibiotic use?* Results.* The worldview-related CAM health concepts enable health promotion oriented infection prevention and treatment aimed at strengthening or supporting the self-regulating ability of the human organism to cope with diseases. There is some evidence that the CAM concepts of health (promotion) are in agreement with current conceptualization of health and that doctors who practice both CAM and conventional medicine prescribe less antibiotics, although selection bias of the presented studies cannot be ruled out. There is some evidence that prevention and some treatment strategies are effective and safe. Many CAM treatment strategies are promising but overall lack high quality evidence.

**Conclusions:**

CAM prevention and treatment strategies may contribute to reducing antibiotic use, but more rigorous research is necessary to provide high quality evidence of (cost-)effectiveness.

## 1. Introduction

Resistance to antibiotics is a complex and growing, international public health problem [1, 2]. Worldwide strategies to control antimicrobial resistance (AMR) and its major consequences (increased mortality, economic impact) are being developed [3, 4]. Currently these strategies appear to be insufficient, as, for example, demonstrated by the unchanged average European consumption rates of antibiotics during the years 2011–2014 [4], although in the UK in 2015 for the first time fewer antibiotics were being prescribed by GPs and clinicians across all healthcare settings than in 2014 [5].

Among others, finding alternatives for antibiotics [2, 6], alone or as part of a delayed prescription approach, may provide a good strategy to optimize appropriate use of antibiotics, meeting both doctors' and patients' needs [7–9]. Alternative nonantibiotic strategies (for symptom relief and/or fighting bacteria) that are currently being studied are, among others, phage therapy, antibodies, immune stimulation, lysins, probiotics, and peptides [2].

At the moment, formal policies advising on the need for alternative strategies to antibiotics do not include the study and/or application of complementary and alternative (CAM) therapies for symptom relief and/or treatment of infections and CAM preventive strategies to reduce the use of antibiotics, although observational studies in Europe have shown that CAM practices and hospitals may have lower antibiotic prescription rates compared to conventional practices [10], due to additional strategies regarding prevention and treatment of infections [11]. In this article we use the term CAM, although elsewhere terms as traditional and complementary medicine [12] or complementary and integrative medicine [13] are used.

Given the mismatch between the urgent need for nonantibiotic strategies and the lack of use of CAM strategies embedded in current conventional policies and clinical practice, we performed a narrative review to determine what is known about the contribution of CAM to help reduce antibiotic use.

## 2. Material and Methods

### 2.1. Research Questions

1. What are the worldview differences between CAM and conventional medicine, relevant for prevention and treatment of infections and the AMR problem?
2. What are the hypothesized CAM contributions to reduce antibiotic use?
3. Is there evidence
  - that supports the proposition that CAM prevention and treatment strategies can lead to the prescription and consumption of fewer antibiotics?
  - that CAM prevention and treatment strategies are effective and safe?

### 2.2. Design

We chose to perform a narrative review based on (1) searches in three databases with a specific, limited set of search terms and (2) input from CAM (research) experts, in order to get a first broad overview of the domain of (possible) contributions of CAM to reduce antibiotic use. Based on the results of this broad narrative review, more methodologically rigorous scoping reviews and/or systematic reviews on subareas of this scientific field can and must be performed.

### 2.3. Identification of Relevant Studies

Searches were performed in PubMed, Embase, and the Cochrane Database of Systematic Reviews (from onset to June 2017).

Search terms used for PubMed and Embase were “health concept”, “prevention AND infection”, “lifestyle AND infection”, “treatment AND infection”, “antibiotic prescription”, “antibiotic consumption”,* each in combination with each of the following search terms for CAM*: “complementary medicine”, “alternative medicine”, “herbal”, “ayurveda OR ayurvedic”, “homeopathy OR homeopathic”, “TCM OR traditional Chinese medicine”, “anthroposophy OR anthroposophic” (for example, “health concept” AND “complementary medicine”; “health concept” AND “alternative medicine”, “health concept” AND “herbal”, etcetera).

Search keywords used for the Cochrane Database of Systematic Reviews were “alternative medicine AND prevention”, “complementary medicine AND prevention”, “complementary medicine AND infection”, “herbal AND infection”, “ayurveda OR ayurvedic”, “homeopathy OR homeopathic”, “TCM OR traditional Chinese medicine”, “TCM OR traditional Chinese medicine AND infection”, “anthroposophy OR anthroposophic”.

### 2.4. Study Selection

The inclusion criteria used in the narrative review were as follows:

- Main research domain:
  - CAM or IM (mandatory)
- Research topics (one research topic is mandatory):
  - Worldview
  - Health (promotion) concept
  - Antibiotic prescription
  - Antibiotic consumption
  - Prevention of infection
  - Treatment of infection

The exclusion criteria in this study were as follows:

- Only conventional prevention or treatment strategies for infections
- CAM/IM for noninfection indications

Study collections were exported to Excel and duplicates were removed. Selection of relevant papers was carried out in two stages and both stages were performed independently by two reviewers. In the first stage, both reviewers read the titles and the abstracts to select potentially relevant papers according to the inclusion criteria. Disagreements were resolved through discussion and consensus with the other review author. In the second stage, the full texts of the included articles were evaluated.

### 2.5. Input from CAM Experts

An international group of CAM clinical and/or research experts was invited for one or two workshops (25/26 January 2017 and 16/17 February 2017) in Frankfurt (Germany), in which, among other things, the scope of the review, barriers and facilitators of the integration of CAM strategies in conventional medicine, and future research activities on the CAM contribution to reduce antibiotic use were discussed. This group and some other CAM experts were also invited to give their input to draft versions of the article.

### 2.6. Analyses

The aim of the qualitative analyses of this narrative review is to map the relevant themes and to provide a first broad overview of the studied domain. As a result, the review does not provide an exact, narrow focused overview of the state of science of each of the subareas (concepts, prevention, treatment per indication) as is done in a scoping review, it does not include all relevant articles if this is not necessary for the mapping purpose, and it does not judge the methodological quality of the scientific evidence of studies on CAM prevention and treatments for specific indications (e.g., assessment of the methodological quality of RCTs with GRADE or of systematic reviews with the AMSTAR 2 checklist), as is done in a systematic review.

## 3. Results

### 3.1. Search Results

See Figure 1.

### 3.2. Worldview Aspects of CAM and Conventional Medicine Relevant for Prevention and Treatment of Infections and the AMR Problem

**Figure 2:**
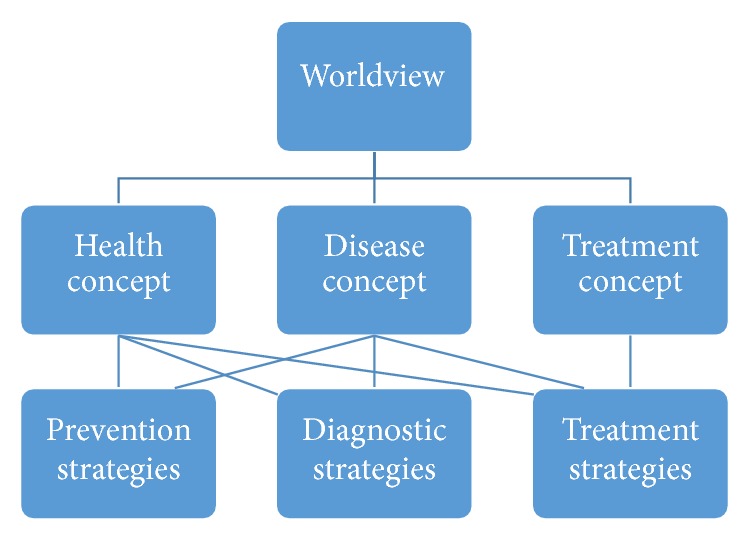
Worldviews, concepts, and clinical practice.

Worldviews are frameworks of meaning and meaning-making that shape how individuals perceive particular issues and their possible solutions and that influence the willingness of individuals to participate in these worldview-related solutions [14]. Medical systems are based on specific, often implicitly handled, worldviews that shape concepts of health, disease, and treatment that in their turn underlie preventive, diagnostic, and treatment strategies applied in clinical practice (Figure 2). A good understanding of the similarities and differences between conventional medicine and CAM worldviews and related concepts, and infection prevention and treatment strategies is expected to contribute to integrate the best of both worlds [15] and is therefore here shortly described.

The main worldview in conventional medicine is the biomedical model. Treatment within this model is mainly oriented at “fighting the disease” both in prevention and treatment, in order to regain the default situation of health [16].

CAM systems (e.g., anthroposophic medicine, ayurveda, homeopathy, traditional Chinese medicine, naturopathy) are whole medical systems, complete systems of theory and practice that have evolved independently over time in different cultures and apart from conventional medicine or western medicine [15, 17, 18]. In daily clinical practice, based on the nonatomistic holistic worldview and related health and disease concepts, CAM stimulates a health promotion oriented lifestyle (prevention) and treats patients with the aim of strengthening or supporting the self-healing or self-regulating ability of the human organism [19] to cope with diseases [20–26].

The differences in worldview and related concepts of health and disease are also expressed in the differences in the main prevention and treatment strategies for infections. Conventional medicine historically was and is (more) focused on fighting disease, more or less implicitly regarding health as the absence of disease. CAM historically focused (more) on health promotion strategies.

The main conventional preventive strategies are vaccinations, hygiene, improving nutrition, and isolation measures [3]. Their aim is respectively to produce immunity and to prohibit contact with microorganisms. The main CAM preventive strategies are lifestyle changes/interventions and medical measures/interventions that strengthen resilience [27]. Their aim is to improve the physiological ability to self-manage and adapt to infections.

The main conventional treatment strategies are antimicrobial treatments that kill or reduce the growth of microbes and reduce disease-related symptoms, like discomfort of fever and pain. The main treatment strategies of CAM are the medicinal and nonpharmaceutical treatments that support the organism to overcome the infection by itself by means of strengthening the self-regulating abilities of the organism (“changing the host's capacities”).

Both in conventional medicine and in CAM, there are currently developments that are aimed at integrating the best of both worlds of fighting disease and health promotion approaches [15, 22].

### 3.3. What Is the Evidence That Supports the Hypothesized CAM Contributions?

#### 3.3.1. Health and Health Promotion Concept

The key elements of the CAM concepts of health are the following: (1) health is the result of a self-regulating inner activity, and (2) health is aimed at restoring wholeness of the organism and balance within or between the functions of body, soul, and spirit [24, 65]. In agreement with the health concept, health promotion can be logically defined as the process of enabling individuals, groups, or societies to increase control over, and to improve, their physiological, psychosocial, and spiritual (meaning in life) health [66]. Health promotion thus aims to improve the development and quality of the self-regulating abilities on these levels and aims to restore balance between opposite functions.

In 1948, the World Health Organization (WHO) defined health as ‘a state of complete physical, mental and social well-being and not merely the absence of disease or infirmity'. In 2011, Huber et al. [67] redefined health as ‘the ability to self-manage and adapt'. This concept is in line with current other concepts of health (e.g., resilience, salutogenesis), emphasizing the role of self-regulating abilities in the physiological, psychosocial, and ‘meaning in life' level as internal resources of the human being to remain or become (more) healthy [22, 68].

One of the mechanisms of acquiring health by self-regulation is the active balancing of opposite functions in the organism, which is increasingly described in the literature on, for example, apoptosis [69] (programmed cell death as an opposite function to ongoing cell division in organisms), wound healing [70], and chronobiology [71]. Imbalances of one of the two opposite functions is related to disease states [72].

In conclusion, we state that there is some evidence that CAM health and health promotion concepts are internally consistent and increasingly in agreement with current health conceptualization in conventional medicine. Their degree of agreement with empirical facts is described in the next paragraphs on health promotion oriented prevention and treatment strategies.

#### 3.3.2. Less Prescription and Consumption of Antibiotics

Several, mostly observational, studies (Table 1) support the hypothesis that practices of doctors who practice both CAM and conventional medicine compared to their conventional colleagues have lower antibiotic prescription rates (measured as past use, antibiotics use ever, in the first 12 months of life and after 12 months of life, consumption, prescription rates) and their patient groups have lower antibiotic consumption rates, although in these studies selection bias (e.g., patients that do not want antibiotics may choose more often a CAM doctor) cannot be ruled out.

#### 3.3.3. Effects and Safety of Prevention Strategies

The main CAM prevention strategies are lifestyle changes/interventions and medical measures/interventions that strengthen and/or support the physiological ability of the organism to self-manage and adapt to infections. Prevention CAM strategies are aimed at (1) reducing stress, insomnia, depression, and anxiety (that are all associated with higher susceptibility to infections), (2) promoting healthy diets and physical exercise (reducing both the risk of infectious diseases), (3) supporting the fever reaction of the organism to infections (to enable the organism to overcome the infection by itself), and (4) preventing infections with natural products.

*Chronic Stress, Insomnia, Depression, and Anxiety Associated with Higher Susceptibility to Acute Infectious Illness. *Chronic stress suppresses or dysregulates innate and adaptive immune responses by altering the Type 1–Type 2 cytokine balance, inducing low-grade chronic inflammation, and suppressing numbers, trafficking, and function of immunoprotective cells. Chronic stress can suppress protective immune responses and/or exacerbate pathological immune responses [73, 74]. Higher reported stress levels [75, 76], short sleep duration (< 6 or 7 hours/night) and poor sleep continuity [77, 78], depression [79], and anxiety [80] are all associated with higher susceptibility to acute infectious illness (e.g., common cold, pneumonia). 

*Chronobiology and General Physiological Recovery.* Chronobiology research has demonstrated that rhythms are present in the whole human organism and all its cells and that they are responsible for the ordered balancing in time between, for example, degenerative and regenerative physiological processes, performance and recovery, sympathetic and parasympathetic activity, work and relaxation, and wakefulness and sleep [71, 81]. Biorhythms are important for recovery in several physiological functions. The speed of recovery differs between biorhythms in the range from very quick recovery (membrane recovery in milliseconds), local tissue recovery in minutes, moderately quick recovery of fatigue by sleeping in 24 hours, until longer recovery periods (vegetative recovery/self-healing (weeks) and trophic/plastic adaptation and growth (months)) [82]. 

*Preventive CAM Strategies.* Several preventive CAM strategies aim to reduce stress, insomnia, depression, and anxiety that are linked to an increased susceptibility to infections [77–80]. CAM prevention includes often promotion of a* rhythmic lifestyle* [71, 82] in order to support general physiological recovery.* Meditation* programs can reduce the negative dimensions of psychological stress [83–87].* Mindfulness* is currently recommended as a useful method for improving mental health and reducing symptoms of stress, anxiety, and depression [88, 89]. Regular* sauna* visits, both in children and adults, reduce the frequency and severity of influenza infections, and the incidence of the common cold [90]. In athletes (intermediate trackers compared to untrained) the immune system is more stimulated with an increased number of white blood cells, lymphocyte, neutrophil, and basophil counts after the sauna session. The main working mechanisms of becoming more resilient (among others to infections) after sauna are optimization of temperature and circulation regulation of skin and mucous membranes, vegetative stabilization with decrease of sympathetic tone (stress reduction), stimulation of nonspecific resistance parameters, and strengthening the antioxidative protection potential and thus the defense against free radicals [91]. Two RCTs demonstrated that* balneotherapy* is beneficial for stress and fatigue reduction in comparison with music or no therapy group [92] and reduces distress by reducing the health risk posed by distress (by 26%), by increasing the health resources (by 11%), and by reducing probability of general health risk (by 18%) [93]. Based on a systematic review of 23 articles [94] it was concluded among others that the administration of various forms of therapeutic* massage* exerted a reduced risk of neonatal sepsis and reduced neonatal stress in very preterm neonates, based on increased vagal activity, increased gastric activity, and increased serum insulin levels. A review of 14 studies on the effects of massage on older people in residential care settings concluded that older people perceive positive effects of massage on factors such as pain, sleep, emotional status, and psychosocial health [95].* Tai-chi* is associated with improvements in psychological well-being including reduced stress, anxiety, depression, and mood disturbance and increased self-esteem [96].* Steiner or Waldorf school education* is associated with lower cortisol levels of children [97–99] and better adjustment to higher education (less anxiety and depression symptoms, greater life satisfaction and academic achievement) compared to children from conventional schools [100]. In Steiner or Waldorf schools, knowledge of biorhythms is applied in the design of the curriculum [101].

Changes of diets are related to rapid changes of the human gut microbiome [102] that is related to the human health and disease status. Currently probiotics, prebiotics, and polyphenols are among the most well established dietary strategies available for modulating either the composition or metabolic/immunological activity of the human gut microbiota [103]. Several “normal”* diet* ingredients are able to positively influence the immune system [104]. A systematic review and meta-analysis with 14 included studies demonstrated that, overall, flavonoid supplementation decreased URTI incidence by 33% (95% CI: 31%-36%) compared with control, with no apparent adverse effects [105]. In a mice model, it was demonstrated that the gut microbiota plays a protective role in the host defense against pneumococcal pneumonia [106]. Polyphenols appear to protect athletes from virus infections following rigorous exercise [107].

A systematic review of 28 articles demonstrated that* exercise* has considerable effects on markers of cellular aspects of the immune system [108]. Current theories regard exercise as a powerful stimulus of immune function [109]. Regular exercise has been shown to improve neutrophil microbicidal functions which reduce the risk of infectious disease, and may be related to improved vaccine responses [110].

*Fever* induction is the result of a fine interplay between the innate immune system and the neuronal circuitry within the central and peripheral nervous systems. It results in the increase of metabolic rate and the enhancement of immune-protective mechanisms (both innate and adaptive) during infection [111]. A small rise in body temperature inhibits bacterial and viral replication (creation of a thermal restriction zone) [111, 112], while at the same time accelerating the immune response (increasing the mobility of polymorphonuclear cells, increasing phagocytosis and T-helper cell adherence, and prevention of lymphocytes cell reduction (CD4 T cells and B cells activity)), and attenuating the immune response/protection against the collateral damage (increased heat shock protein causing a decrease of NF-*κ*B, reduced TNF*α*, and reduced IFN*γ*) [113]. In men, early acute respiratory distress syndrome (ARDS), an elevated peak temperature in the first 24 hours in ICU in critically ill patients with an infection [114], is associated with improved survival rates (adjusted OR: 0.56, 95% CI: 0.48–0.66). It is now evident that antipyretic treatment (paracetamol, aspirin, or ibuprofen) does not prevent seizures [115]. A recent review and meta-analysis demonstrated that antipyretic treatment does not prolong the fever or illness, but may alter inflammatory processes, especially in the early phase during which the immune response develops. It may lead to a reduction of the initial adaptive response. Antipyretic treatment has been shown to increase the spread of infection and prolong influenza, chicken pox, and common colds at the population level and may increase both the rate and duration of viral shedding, further increasing the pathogen's transmission rate; this effect has been shown experimentally for influenza in ferrets. A higher transmission rate in general will lead to larger epidemics and hence to greater morbidity and mortality [116].

Finally, prevention of infections is achieved by use of natural products, for example, prevention of wound and gastrointestinal infections by apitherapy and respiratory tract infections by probiotics [53, 117, 118].

#### 3.3.4. Effects of Treatment Strategies

Evidence of the effects of CAM medicinal treatment strategies comes from 12 Cochrane reviews (Table 2), 16 non-Cochrane reviews (Table 3), 15 clinical studies (Section 3.3.4 (2)), and 20 studies on traditional use and in vitro studies (Section 3.3.4 (3)). Systematic reviews were categorized per indication (respiratory tract infections (Cochrane reviews (CRs): 7, Non-Cochrane reviews (NCRs): 13); urinary tract infections (CRs: 2, NCRs: 1); and other infections (CRs: 3, NCRs: 0). In addition the results of two NCRs on antibiotic-associated diarrhoea were described. Clinical studies were categorized per indication: acute respiratory and ear infections (observational studies: 2), otitis media (RCT:1, observational study: 1), infected wounds and MRSA (RCTs: 4), and other infections (RCTs: 7, observational studies: 2).

The Cochrane and non-Cochrane reviews demonstrate that some CAM treatment strategies for respiratory infections (both children and adults) are promising and that some have been shown to be effective in systematic reviews. CAM treatment strategies for other infections such as urinary tract infections (adult women) and skin infections are promising, but more rigorous research is necessary to provide high quality evidence. 

*(1) Other Non-Cochrane Reviews.* See Table 3.

*(2) Individual Clinical Studies.* There are many other CAM treatments for infections which have been studied in a clinical trial, but for which no systematic review has yet been completed. 

*Acute Respiratory and Ear Infections.* An international, multicenter, cohort study, comparing homeopathic and conventional treatment of acute respiratory and ear complaints in a primary care setting with 1.577 patients (857 received homeopathic and 720 conventional treatment), demonstrated that homeopathic treatment was not inferior to conventional treatment. More statistically significant favorable results for homeopathy were as follows: onset of improvement within the first 7 days after treatment was significantly faster upon homeopathic treatment both in children and adults, and adverse drug reactions occurred more frequently in adults of the conventional group than in the homeopathic group [119]. A prospective observational study comparing anthroposophic (AM) and conventional treatment of children with acute respiratory or ear infections under routine primary care conditions demonstrated that AM treatment was associated with much lower use of antibiotics (5% vs. 26%, during the four-week follow-up) and also much lower use of analgesics/antipyretics (3% vs. 26%) and was safe. AM patients demonstrated somewhat quicker symptom resolution and higher caregiver satisfaction [32]. 

*Otitis Media.* There is some evidence that* Juzen-taiho-to*, a Kampo or traditional Japanese herbal medicine, is effectively preventing recurrent acute otitis media (AOM) in children [120]. A prospective nonrandomized, comparative study underlined these results in the treatment of children with chronic otitis media with effusion. The frequency of antibiotic use was significantly less with the integrative concept using integrative-anthroposophic treatment (17.9% vs. 82.9%) [121]. 

*Infected Wounds and MRSA. Tea Tree Oil* (TTO) is an essential oil derived mostly from the leaves and terminal branchlets of the Australian native plant Melaleuca alternifolia from the Myrtaceae family [122]. Although no Cochrane systematic review has been conducted yet with regard to effects of TTO on* MRSA* infections, there is low quality evidence that 10% TTO soap significantly reduces MRSA quantity in infected wounds. TTO has been studied as an alternative treatment option for* MRSA *infections [123–126]. In a RCT a 10% TTO cream and 5% TTO body wash were more effective in clearing* MRSA* on skin compared with 4% chlorhexidine gluconate soap and 1% silver sulfadiazine cream [124]. 

*Other Infections.* There is some evidence that CAM use in patients with cancer is associated with a reduction in hospitalizations and requirements for antibiotics [127]. Trials of complementary interventions (vitamin A, probiotics, cranberry, nasturtium, and horseradish) for prevention of recurrent urinary tract infection in children generally gave favorable results but were not conclusive [128]. Herbal therapies appeared to be at least as effective as rifaximin for resolution of small intestine bacterial overgrowth and as effective as triple antibiotic therapy for SIBO rescue therapy for rifaximin nonresponders [129]. Long-term vaginal administration of* Lactobacillus rhamnosus* appears to be a useful complementary approach in the management of bacterial vaginosis [130]. A RCT, comparing the effects of vaginal cream with* thyme* and* garlic* and Metronidazole vaginal gel on treatment of bacterial vaginosis, demonstrated that both treatments were equally effective [131]. 

*(3) Traditional Use and/or In Vitro Studies.* Many CAM treatments have not been studied (at all or not for specific indications) in clinical studies with humans. They have only a status of long traditional use and/or have demonstrated antimicrobial effects in vitro. We describe here a very small selection of examples without any claim on completeness in selecting the examples. The importance of describing them here is that, given the urgent need for alternative, nonantibiotic treatments, this group of CAM treatments might be a source of development of new nonantibiotic alternatives. Positive experience during long periods of traditional use and/or positive results of in vitro studies provide a reason to study these remedies in clinical studies with patients.

*Anemarrhena asphodeloides* had been used in China, Japan, and Korea for thousands of years and demonstrates both antimicrobial and antiviral activities in vitro [132, 133].* Asparagus racemosus* might be an alternative to antibiotics during UTI infections [134, 135].* Diodia scandens* and* Phyllanthus amarus* might be effective against* MRSA* [136], extract 220D-F2 from the root of* Rubus ulmifolius* can be used to inhibit* S. aureus* biofilm formation to a degree that can be correlated with increased antibiotic susceptibility without toxic effects on normal mammalian cells [137],* Woodfordia fruticosa* appears to be effective against* Pseudomonas pseudoalcaligenes* and Gram-negative bacteria [138, 139], a review identified 255 (70% of 365) plant species from a wide range of families that have shown antimycobacterial activity [140], and protocatechuic acid (PCA, 3,4-dihydroxybenzoic acid) is a phenolic compound found in many food plants that demonstrates antimicrobial activities and also exerts synergistic interaction with some antibiotics against resistant pathogens [141].* Asparagus racemosus* demonstrated positive effects in vitro against several UTI Gram-negative and Gram-positive pathogens [135]. Apitherapy is used in many traditional medical systems, among others, for the prevention of respiratory infections [142]. Natural honey demonstrated antiacanthamoebic properties [143], antibacterial effects [144], and antibiofilm effects against* S. aureus* [145] in wound healing.* Melissa officinalis L.* demonstrates both antibacterial and antifungal effects [146]. Several studies describe a list of promising CAM treatments for several infections, based on clinical experience and/or in vitro studies [147–150].

*Andrographis paniculata* has already been shown in clinical trials to be effective for respiratory infections (see above). In addition to this it may be effective against other infections. It is currently used in ayurveda, homeopathy [151], and TCM [152]. In vitro studies demonstrated dose-dependent antibacterial effects of* A. paniculata*: against* E. coli, Klebsiella, Staphylococcus *and* Pseudomonas [153], Salmonella, Shigella, Gram A Streptococci, S. aureus, MRSA, Pseudomonas aeruginosa [154], Salmonella typhimurium, E. coli, Shigella sonnei, Streptococcus pneumonia, Streptococcus pyogenes, Legionella pneumophila, *and* Bordetella pertussis* [155]. However in one study no activity against* E. coli* or* Klebsiella pneumoniae* was detected [154].

*Pelargonium sidoides* has already been tested in clinical trials for respiratory infections (see above) but may also be helpful in the treatment of other infections. Its traditional use was for the treatment of diarrhoea. Mechanistic evidence from a review in 2014 [156] concluded the following: ‘experimental results from in vitro studies indicate that bioactive phytochemical constituents of* Pelargonium sidoides* may not possess a direct antimicrobial effect, but instead act by interfering with microbial binding to host cell receptors, inhibition of key enzymes and the production of antimicrobial effector molecules such as nitric oxide and interferons (IFNs) by the host cells.' However, other in vitro studies did demonstrate growth inhibition of* Escherichia coli, Shigella sonnei, Staphylococcus aureus OK2a *and* S. aureus ATCC6538, Salmonella typhi, S. typhimurium, Shigella flexneri, *and* Staphylococcus aureus OK2b* [157].

Several studies also demonstrate the potential of treatment with natural products of viral infections, for example, licorice for several viral infections [158].

*(4) Nonpharmaceutical Interventions.* There is some evidence that acupuncture is effective in pain reduction of acute sore throat [159, 160]. Blue light demonstrates bactericidal effects in vitro and in vivo [161–163]. Based on the expertise of anthroposophic nurses, there is some practice-based evidence that external applications with essential oils may have effects on symptom relief [164].

#### 3.3.5. Safety

General side effects of antibiotics use are antibiotic-associated diarrhoea (AAD) that happens in 5-39% of patients that are prescribed antibiotics [63] and candidiasis, obesity (associated with childhood use of antibiotics before 2 years) [165–169], allergies (in 5-10% of all patients [170] and in 10-30% of hospital patients [171]), increase of irritable bowel syndrome (IBS), and irritable bowel disease (IBD) symptoms [172]. In the treatment of AOM for every 14 children treated with antibiotics, one child experienced an adverse event (such as vomiting, diarrhoea, or rash) that would not have occurred if antibiotics had been withheld [173].

According to a systematic review of results reported in RCTs with 111 studies of a single herb and 133 of multiple herbs with a total of 15,441 participants, herbal treatment may be considered as safe: ‘There were 480 cases (3.1%) of adverse events (344 for single, 136 for multiple herb studies; p < 0.01). A total of 259 cases reported blood test abnormalities, including five cases of abnormality in hepatic functional enzymes. The most frequently reported adverse event was digestive symptoms (44.3%), followed by nervous system symptoms (17.3%) and behaviors such as loss of appetite (16.3%).' [174] However, a few herbal treatments have been associated with severe adverse events [175–177]. In reaction to these problems, pharmacovigilance systems have been established in the main producing countries of Chinese herbals [178] and Ayurvedic herbals [179], and quality standards for these medicinal products (MPs) have been improved [180, 181]. Other concerns are the inadequate knowledge of their mode of action, potential adverse reactions, contraindications, and interactions with existing orthodox pharmaceuticals and functional foods [182, 183].

Adverse reactions to homeopathic and anthroposophic MPs are infrequent and usually of mild to moderate severity, and anaphylactic reactions occur but are very rare [15, 57, 184].

## 4. Discussion

Given the mismatch between the urgent need for nonantibiotic strategies and the lack of use of CAM strategies embedded in current conventional policies and clinical practice, we performed a narrative review, based on searches in three databases (PubMed, Embase, and the Cochrane Database of Systematic Reviews) with a specific, limited set of search terms and input from CAM (research and clinical) experts, to explore and map what is known about the contribution of CAM health and health promotion concepts, infection prevention, and infection treatment strategies to reduce antibiotic use. This review has found significant evidence to support the safety and effectiveness of a range of CAM treatments for respiratory infections, based on many systematic reviews. It is now important to assess how this information can be used to recommend alternatives to antibiotics and so avoid unnecessary use of antibiotics in clinical practice. For other types of infection (such as urinary infections and skin infections) there are some promising clinical trials, but more research is needed before recommendations can be made.

### 4.1. Strengths and Limitations

The main strength of this review is the broad overview on this domain: the differences between conventional medicine and CAM regarding worldview, health (promotion) concepts, related prevention and treatment strategies for infections, and supporting evidence. This broad scope may contribute to providing a more transparent view on the differences between conventional medicine and CAM and on the possible contribution of CAM strategies. The promising results may also provide a broader interest in conventional medicine to study the contribution of CAM to the reduction of antibiotic use, and provide interest in the professional integration of the best of both worlds of CAM and conventional medicine in prevention and treatment strategies of infections, in line with, for example, the current ‘Traditional Medicine Strategy: 2014-2023' of the World Health Organization (WHO) [12].

The review has also several limitations. The first one is that it is a narrative review that is aimed at exploring relevant themes and at providing a first broad overview of the studied domain. The review thus does not provide an exact, narrow focused overview of the state of science of each of the subareas (concepts, prevention, treatment per indication) as is done in a scoping review, and it does not judge the methodological quality of the scientific evidence of studies on CAM prevention and treatments for specific indications, as is done in a systematic review. A second limitation is that the number of databases searched was limited to three (PubMed, Embase, Cochrane Database of reviews), that a limited number of search terms was used, that the acquired additional records were not collected in a systemized way, and that input was given by a selected group of CAM research and/or clinical experts, which might have led to a selection regarding the content provided as input. A third limitation is that the quality of the studies was often low. For example, many of the studies on prescription of antibiotics (may have) used self-selected samples. It is therefore not obvious that it would be possible to reduce use of antibiotics simply by transferring patients from conventional to integrative doctors, because the reason may be that patients self-selecting to go to CAM doctors are less likely to demand antibiotics.

### 4.2. Medical and Methodological Barriers and Facilitators of the Implementation of CAM Strategies into Conventional Medicine

Besides, in many cases, the absence of high quality evidence, the implementation of CAM prevention and treatment strategies into conventional medicine is hindered by several other medical and methodological barriers.

Whereas CAM modalities were tolerated in clinical practice in many western countries until the end of the 20th century, they are increasingly becoming scientifically criticized [15, 185]. According to many scientists, CAM treatments are justified with prescientific or unscientific paradigms that are not in agreement with currently accepted medical theories. Therefore, on theoretical grounds, it is their and others' opinion that CAM must not be integrated with conventional medicine [186–188]. A second group of barriers concerns the quality of CAM prevention and treatment strategies. The image of CAM, in conventional science and medicine, is often that there is no high quality evidence of specific effects of CAM strategies for conventional indications as tested in clinical studies and analyzed in systematic reviews and meta-analyses [186]. In addition, there are concerns regarding the safety and drug interactions of CAM treatments [189–191], because some medicinal products have been associated with repeated, severe adverse reactions [192, 193]. Other concerns are environmental contaminations (e.g., air pollution, soil contaminations), cultivation practices (e.g., pesticides, fungicides, microorganisms, endotoxins), manufacturing procedures (e.g., microorganisms, endotoxins), and inappropriate use [194, 195].

Another group of barriers concerns implementation of CAM strategies. First, whereas some conventional guidelines are still based on clinical expertise and/or lower level of evidence (e.g., in pediatrics and surgery), input for guidelines from CAM based on clinical expertise and/or lower level of evidence is not easily accepted, based on the described negative image of CAM. Secondly there are regulatory barriers. In essence, although European regulatory systems are available for homeopathy and herbal medicines, they currently do not match the specific features of whole medical system products with regard to assessment of quality [196], effectiveness and safety, and handling of multicomponent products [15]. Thirdly, the available evidence and knowledge on prevention and treatment strategies are not easily accessible for the target populations (e.g., doctors, pharmacists, patients) [197]. Fourthly, although many patients already use CAM MPs [198, 199] and shared-decision making is promoted in clinical practice, many doctors do not want to prescribe CAM alternatives for antibiotics, due to patient pressure to prescribe antibiotics [200–202], fear of ineffectiveness of CAM treatments [203], lack of knowledge on CAM in general [204], insufficient information on effectiveness and safety, (assumed) insufficient regulation of herbal practitioners, concerns about herbal quality control and potential herb–drug interactions [205, 206], and a lack of communication between doctors and patients about this topic [204, 207].

An important methodological barrier for CAM is that methodologies that are currently used to acquire high quality evidence (RCTs, systematic reviews, and meta-analyses of RCTs) often do not match CAM, so-called whole medical system, interventions. The current golden standard of EBM, the double-blind, placebo-controlled RCT, is often not applicable to test efficacy and effectiveness of a CAM intervention due to its complexity [208, 209]. Furthermore there are significant regulatory barriers to conducting clinical trials of complex or individualized mixtures of herbal medicines. CAM treatments for conventional indications are often individualized based on additional CAM diagnoses, are aimed at restoring balances rather than symptom reduction, often contain different treatments as part of a complex intervention (multimodal), and are system effects and health promotion oriented. As a result of this mismatch between demanded methodologies and CAM interventions, there is a lack of RCTs. And the available RCTs with protocolled interventions might lead to false-negative results (meaning that in reality the treatment has (larger) beneficial effects but these are not captured in the research study), because of the lack of individualization. Therefore, CAM researchers argue that there is a need for additional methods, e.g., pragmatic studies, observational studies, a mix of qualitative and quantitative studies, and n=1 studies, in order to meet the complexity of CAM interventions [210]. In addition, a “reversed research strategy” for assessing CAM has been suggested, starting with studies of the context, paradigms, philosophical understanding, and utilization, then subsequently the safety status of the whole system, comparative effectiveness of the whole system, specific efficacy of components, and finally the underlying biological mechanisms [78, 107]. A second barrier is that there is a lack of structural funding of research on CAM prevention and treatment strategies in many countries (although in China the research on TCM is increasingly structurally funded), with a sponsorship bias as a result [211]. Most of CAM is not patentable and not profitable, has little lobby, and is complicated and multifactorial, and therefore research is underfunded.

On the other hand, there are also several facilitators for the integration of CAM prevention and treatment strategies for infections. First of all, the position of the patient in healthcare is increasingly important (as expressed in developments like, for example, shared-decision making, patient reported outcomes, and experiences) and CAM is increasingly used and demanded by patients worldwide [43, 207, 212]. Secondly, the increasing burden of the global AMR problem opens opportunities for CAM alternative prevention and treatment approaches as expressed in a NHS funded study on CAM treatment of UTI [213] and a EU funded European research network for CAM researchers on infections [214]. Thirdly, the CAM concept of health promotion is increasingly in agreement with conceptualization of health in conventional medicine [66, 67, 215]. CAM prevention and treatment health promotion strategies are thus not, as often assumed, justified with a prescientific or unscientific paradigm, but are based on theories that are increasingly accepted in conventional medicine. Fourthly, there is a growing scientific interest in and knowledge of systems biology/systems and personalized approaches in conventional science and medicine, which makes it easier to accept the CAM systems approaches [22]. Fifthly, there are positive examples of the integration of CAM strategies, especially in low-income countries [216], that can result in more confidence in CAM. Sixthly, there is an increasing interest in the delayed prescription strategy, which fits with the CAM treatment strategy; during the delayed prescription period patients can use CAM treatments. Reduced antibiotics prescription for uncomplicated infections, without additional CAM treatment, is already relatively safe with only a very slight increase in the incidence of complicated infections [217, 218]. In addition, suggesting actions parents could take to reduce their child's symptoms (providing parents positive treatment recommendations, such as CAM treatments) is associated with decreased risk of antibiotic prescribing [219]. Last but not least, there are some good examples of positive results from CAM prevention and treatment strategies of infections with high quality evidence from systematic reviews/meta-analyses [51, 59, 63].

### 4.3. Future Research and Other Nonresearch Perspectives

The narrative review provides concrete leads for future research and other activities. First, in general this narrative review must be followed by scoping reviews and/or systematic reviews, examining each of the subareas (e.g., prescription rates of antibiotics) separately, providing a more complete overview of the subarea (scoping review) and a better judgment of the quality of the scientific evidence (systematic review).

Although this first narrative review on CAM approaches to reduce antibiotic use, based on a systematic search strategy, is limited and not yet a systematic review of the literature, it can help to structure directions and types of further research. The following topics seem to be important for future research in the domains that were studied here (health and health promotion concepts, antibiotic prescription and consumption, safety and effects of prevention and treatment strategies). Regarding the CAM health (promotion) concepts, more studies validating the health concept and exploring and testing the health promotion working mechanisms (in general and specific regarding infections) are necessary. With regard to the comparison of prescription and consumption rates of antibiotics in CAM practices and conventional practices, more studies are needed in other integrative primary and secondary care facilities, studies on specific indications, and better studies controlling for selection bias. With regard to the safety and effects of CAM prevention and treatment strategies, more research is needed regarding the effects and safety of CAM lifestyle/prevention strategies on the development of resilience to infections; nosocomial infectious diseases and resistance rates, hygiene management in different types of hospitals (CAM vs. conventional); and the identification, collection, and/or systematization of CAM expert knowledge based on traditional use.

Regarding treatment, further research building on current high levels of evidence could focus on use of CAM as alternatives to antibiotics for respiratory infections. This research and respective methodologies could build on the existing systematic reviews. Decision aids and guidelines need to be developed, piloted, and evaluated, to guide clinicians and patients in their choice of CAM therapies. Economic analyses will also be important to guide policy development in this area.

For other disease areas, there is as yet insufficient evidence for the development of guidelines or decision aids, so more research is needed to build the evidence base with this goal in mind. There is interesting preliminary evidence from systematic reviews on use of CAM for treatment of urinary tract infections and prevention of recurrent UTIs. Further rigorous research is needed in order to find the optimal CAM treatments for these conditions, as alternatives for antibiotics. Another important area is the treatment of antibiotic-resistant skin infections, such as wounds infected with MRSA. There are some preliminary clinical trials, but no systematic reviews in this area. Many antibiotics are prescribed for skin infections, especially acne, yet our search did not identify any RCTs of CAM treatments for acne. Systematic reviews and more rigorous clinical trials are needed to find the most effective CAM approaches for these conditions.

In many countries worldwide a wide range of CAM treatments are used daily but not well observed by the scientific community. Such research and knowledge gaps as well as low levels of evidence can derive from low research capacity and /or methodological challenges. They may be relevant for further research and for public health policy. AMR public health policy will become more interested in learning about the potential contribution of CAM to reduce prescription of and demand for antibiotics. Preliminary research may serve as a starting point with respect to some of these fields of current CAM practice, to highlight the most promising therapies, for example, through observational studies such as retrospective treatment-outcome studies [220].

Finally, according to the group of CAM experts, other issues, supporting the study of the CAM contribution and the integration of the contribution of CAM into conventional medicine, should be addressed in future activities: adequate research infrastructure should be developed and optimized (e.g., (academic) institutes, methodologies, funding) to test CAM multicomponent and/or multimodal treatments of infections; adequate regulatory infrastructures should be developed to regulate multicomponent and/or multimodal CAM treatments of infections; adequate information on the CAM contributions needs to be provided for different stakeholder groups (e.g., doctors, pharmacists, patients), through the development of best practices, decision support tools, training, communication, and implementation strategies including proposals for the integration of CAM/IM contributions in guidelines. There need to be more studies of patients' demands, decision criteria, and awareness of side effects of antibiotics and both patients' and doctors' perceptions on antibiotics and the AMR problem. There is a need for cross-country analyses of socioeconomic factors, insurance policies and regulation with regard to antibiotic prescription, effectiveness of public health policies, and implementation of guidelines as well as CAM prevention and treatment strategies.

## 5. Conclusions

1. There is some evidence that CAM prevention and treatment strategies can lead to the prescription and consumption of fewer antibiotics.
2. There is some, most often low quality, evidence that CAM prevention and treatment strategies are safe and effective (reduction of incidence of recurrent infections, overall symptoms and specific symptoms of infections, symptom severity, and time to recovery/sick leave).
3. Some of the CAM treatment strategies for respiratory infections are promising and some have been shown to be effective in systematic reviews. Guidelines and decision aids are needed for patients and clinicians.
4. CAM treatment strategies for other infections such as urinary tract infections and skin infections are promising, but more rigorous research is necessary to provide high quality evidence before guidelines can be developed.
5. The worldview differences between CAM and conventional medicine, relevant to prevention and treatment of infections and the AMR problem, are the differences between the biomedical model and the whole medical system model.
6. The worldview-related CAM health concepts enable health promotion oriented prevention and treatment of infections aimed at strengthening or supporting the self-regulating ability of the human organism to cope with diseases.
7. The hypothesized CAM contributions to the reduction of antibiotic use are
  - prevention strategies aimed at reducing stress, insomnia, depression, and anxiety (all associated with increased susceptibility to acute infectious illness), promoting healthy diets and physical exercise (both reducing the risk of infectious diseases), supporting the fever reaction of the organism (to overcome the infections by itself), and preventing infections with natural products;
  - treatment strategies with natural medicinal products.

## Figures and Tables

**Figure 1:**
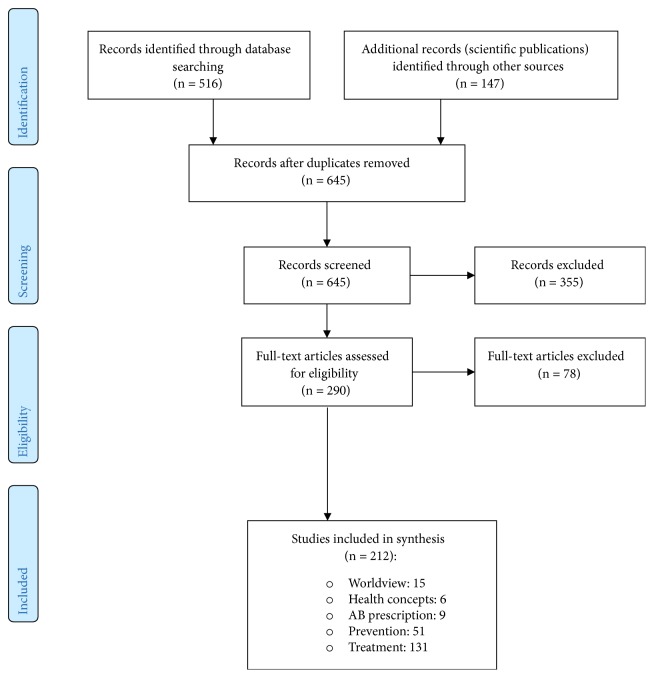


**Table 1:** Studies on prescription and consumption rates of antibiotics in CAM practices and in families with an alternative lifestyle.

Study type	Results	Study characteristics
Cross-sectional study comparing children from anthroposophic families and children with a non-anthroposophic lifestyle [28]	Past use of antibiotics: (i) anthroposophic children: 52% (ii) non-anthroposophic children: 90% (odds ratio (OR): 0.62, 95% CI: 0.43 - 0.91)	N = 295 anthroposophic children and 380 non-anthroposophic children, age 5-13 years. Sweden

Cross-sectional study comparing children from anthroposophic families and children with a non-anthroposophic lifestyle [29]	Antibiotics use ever, in the first 12 months of life and after 12 months of life: (i) all significantly lower in children with an anthroposophic lifestyle (p < 0.001)	N = 6.630 children, age 5-13 years (4.606 from Steiner schools and 2.024 from reference schools) in 5 European countries (Austria, 11%; Germany, 39%; The Netherlands, 22%; Sweden, 9%; Switzerland, 20%)

KOALA Birth Cohort Study comparing families with an alternative and a non-alternative lifestyle [30]	Families with an alternative lifestyle: (i) antibiotic use was less frequent (13.8% vs. 24.1%) (p-value not presented)	N= 2.343 conventional children and 491 alternative lifestyle children. The Netherlands

Observational study on prescribing practices of anthroposophic medicine (AM) doctors in the treatment of upper respiratory tract infections [31]	Prescription rate for antibiotics (6.3%) was well below the German average	21.818 prescriptions for 12.081 patients (73.7% children) with 19.050 cases of URTI were analysed. Antibiotics were given in 6.3% of cases (minimum: common cold 1.9%, maximum: tonsillitis 24.3%). Germany

Prospective, non-randomised comparison of outcomes in patients self-selected to anthroposophic or conventional therapy under real-world conditions [10]	5.5% of the patients in the AM group and 33.6% in the conventional group received antibiotics (p < 0.0001)	29 primary care practices (Austria, Germany, Netherlands, UK and USA). N= 1.016 outpatients, age ≥ 1 month, consulting an anthroposophic (N = 715) or conventional physician (N = 301) with a chief complaint of acute (≤ 7 days) sore throat, ear pain, sinus pain, runny nose or cough

Prospective, non-randomised comparison of outcomes in patients self-selected to anthroposophic or conventional therapy under real-world conditions [32]	5.5% of the patients in the AM group and 25.6% in the conventional group received antibiotics (p < 0.001)	N = 529 children <18 years from Europe (Austria, Germany, Netherlands, and UK) or USA with acute respiratory or ear infections

Observational study on the treatment of patients with upper respiratory tract infections: homeopathic GPs vs. conventional GPs [33]	Significantly lower consumption of antibiotics (OR* *=* *0.43, 95% CI: 0.27-0.68) in the homeopathic patients group	N = 518 adults and children with URTI (79.3% rhinopharyngitis). France

Randomized trial, children 6 months to 11 years old, diagnosed with AOM and managed with a delayed antibiotic approach, randomized to standard therapy alone or standard therapy plus a homeopathic ear drop preparation [34]	Significantly less antibiotic use in the homeopathic group (26.9% vs. 41.2%) (p-value not presented)	N = 456 patient visits were compared: 281 received homeopathy, 175 received conventional medicine. Germany, Switzerland, Austria, USA

Observational study among parents of children [35]	Use of homeopathic products not associated with decreased antibiotic consumption (adjusted OR = 1.02, 95% CI: 0.84 - 1.24).	N = 9.723 parents of children, age: 3–4.5 years. United Kingdom

**Table 2:** Cochrane reviews of CAM treatments of infections.

Treatment and indication	Main conclusions	Study characteristics
**Respiratory tract infections (RTIs)**		

Immunostimulants (IS) (including herbal treatments) for preventing respiratory tract infection in children [36]	IS reduce the incidence of acute RTIs by 40% on average in susceptible children Further RCTs are required	Thirty-five placebo-controlled trials (N = 4.060). The use of IS was shown to reduce ARTIs measured as the total numbers of ARTIs (MD -1.24; 95% CI: -1.54 to -0.94) and the difference in ARTI rates (MD -38.84%; 95% CI: -46.37% to -31.31%).

Oral *Astragalus* (*Huang qi*) for the prevention of frequent acute respiratory tract infections in children [37]	Insufficient evidence of the effectiveness and safety	No studies met the inclusion criteria

*Garlic* for prevention of the common cold [38]	There is insufficient clinical trial evidence Further RCTs are required	Only one trial met the inclusion criteria. N= 146 participants. Interventions: either a *garlic* supplement (with 180 mg of allicin content) or a placebo (once daily) for 12 weeks. Results: 24 occurrences of the common cold in the *garlic* intervention group compared with 65 in the placebo group (p < 0.001), resulting in fewer days of illness in the garlic group compared with the placebo group (111 versus 366). The number of days to recovery from an occurrence of the common cold was similar in both groups (4.63 versus 5.63).

*Echinacea* for the common cold [39]	There is possibly a weak benefit from some *Echinacea* products	Twenty-four double-blind trials with 4.631 participants including a total of 33 comparisons of *Echinacea* preparations and placebo met the inclusion criteria. None of the 12 prevention comparisons reporting the number of patients with at least one cold episode found a statistically significant difference. However a post hoc pooling of their results suggests a relative risk reduction of 10% to 20%. Of the six treatment trials reporting data on the duration of colds, only two showed a significant effect of *Echinacea* over placebo.

*Pelargonium sidoides* for acute rhinosinusitis, the common cold and acute bronchitis [40]	*P. sidoides* may be effective in alleviating symptoms of acute rhinosinusitis and the common cold in adults, but doubt exists. It may be effective in relieving symptoms in acute bronchitis in adults and children, and sinusitis in adults The overall quality of the evidence was considered low for main outcomes in acute bronchitis in children and adults, and very low for acute sinusitis and the common cold	Of 10 eligible studies, eight were included in the analyses; two were of insufficient quality. Three trials (746 patients, low quality of evidence) of efficacy in acute bronchitis in adults showed effectiveness for most outcomes in the liquid preparation but not for tablets. Three other trials (819 children, low quality of evidence) showed similar results for acute bronchitis in children. One study in patients with sinusitis (n = 103 adults, very low quality of evidence) showed significant treatment effects (complete resolution at day 21; RR 0.43, 95% CI: 0.30-0.62). One study in the common cold demonstrated efficacy after 10 days, but not five days (very low quality of evidence).

Chinese herbals for sore throat [41]	Some Chinese herbal medicines appeared efficacious Due to methodological weaknesses no final conclusions could be drawn	12 studies involving 1.954 participants. Ten studies were identified as being of methodologically poor quality and two studies as being of medium quality. No meta-analyses. Six formulations were shown to be superior to the control in improving recovery: *Ertong Qingyan Jiere Koufuye* was more effective than *Fufang Shuanghua Koufuye* for acute pharyngitis (odds ratio (OR) 2.52; 95% CI: 1.11-5.74); *Yanhouling* mixture was more effective than gentamicin atomised inhalation for acute pharyngitis (OR 5.39; 95% CI: 2.69-10.81); *Qinganlan Liyan Hanpian* was more effective than *Fufang Caoshanhu Hanpian* for acute pharyngitis (OR 2.25; 95% CI: 1.08-4.67); sore throat capsules were more effective than antibiotics (intravenous cefalexin) for acute pharyngitis or acute tonsillitis (OR 2.36; 95% CI: 1.01-5.51); compound dandelion soup was more effective than sodium penicillin for acute purulent tonsillitis (OR 5.06; 95% CI: 1.70-15.05); and eliminating heat by nourishing yin and relieving sore-throat methods combined with *Dikuiluqan Hanpian* were more effective than *Dikuiluqan Hanpian* alone for children with chronic pharyngitis (OR 2.63; 95% CI: 1.02-6.79). Another six formulations were shown to be equally efficacious as the control.

Chinese medicinal herbs for acute bronchitis [42]	There is insufficient quality data	None of 74 studies involving 6.877 participants met the inclusion criteria.

**Urinary tract infections (UTIs)**		

Chinese herbal medicine (CHM) for recurrent urinary tract infections [43]	CHM as an independent intervention or in conjunction with antibiotics may be beneficial for treating recurrent UTIs during the acute phase of infection and may reduce the recurrent UTI incidence for at least six months post-treatment Better quality evidence is needed	Seven RCTs involved a total of 542 women; of these, five recruited post-menopausal women (aged from 56 to 70 years) (422 women). All studies were assessed to be at high risk of bias. Analysis of three studies involving 282 women that looked at CHM versus antibiotics suggested that CHM had a higher rate of effectiveness for acute UTI (RR 1.21, 95% CI: 1.11–1.33) and reduced recurrent UTI rates (RR 0.28, 95% CI: 0.09-0.82). Analysis of two studies involving 120 women that compared CHM plus antibiotics versus antibiotics alone found the combined intervention had a higher rate of effectiveness for acute UTI (RR 1.24, 95% CI: 1.04-1.47) and resulted in lower rates of recurrent infection six months after the study (RR 0.53, 95% CI: 0.35-0.80). One study comparing different CHM treatments found *Er Xian Tang* was more effective in treating acute infection in post-menopausal women than *San Jin Pian* (80 women: RR 1.28, 95% CI: 1.03-1.57). Analysis showed that active CHM treatments specifically formulated for recurrent UTI were more effective in reducing infection incidence than generic CHM treatments that were more commonly used for acute UTI (RR 0.40, 95% CI: 0.21-0.77).

Probiotics for preventing urinary tract infections in adults and children [44]	There is insufficient quality data	Nine studies involved 735 people. Four studies compared probiotic with placebo, two compared probiotic with no treatment, two compared probiotics with antibiotics in patients with UTI, and one study compared probiotic with placebo in healthy women. All studies aimed to measure differences in rates of recurrent UTI. Overall, there was a high risk of bias in the included studies. No significant reduction in the risk of recurrent symptomatic bacterial UTI was found between patients treated with probiotics and placebo (6 studies, 352 participants: RR 0.82, 95% CI: 0.60-1.12; I2 = 23%). No significant reduction in the risk of recurrent symptomatic bacterial UTI was found between probiotic and antibiotic treated patients (1 study, 223 participants: RR 1.12, 95% CI: 0.95-1.33).

**Other infections**		

Chinese medicinal herbs for preventing infection in nephrotic syndrome [45]	A compound of Chinese medicinal herbs—*Tiaojining*—may have positive effects on prevention of nosocomial or unspecified infection with no obvious serious adverse events in children with nephrotic syndrome Better quality evidence is needed	Twelve studies conducted in China, including 762 children with nephrotic syndrome were identified. No studies were identified in adults. All studies compared one kind of prophylactic pharmacotherapy (intravenous immunoglobulin (IVIG), thymosin, oral transfer factor, mannan peptide tablet, Bacillus Calmette-Guerin (BCG) vaccine injection, polyvalent bacterial vaccine (Lantigen B) and two kinds of Chinese medicinal herbs: a compound of Chinese medicinal herbs (*Tiaojining*) and *Huang qi (astragalus)* granules) plus baseline treatment with baseline treatment alone. No RCTs were identified comparing antibiotics, non-pharmacological prophylaxis, or pneumococcal vaccination. Four studies showed a significantly beneficial effect of IVIG on preventing nosocomial or unspecified infection in children with nephrotic syndrome (RR 0.47, 95% CI: 0.31-0.73). Thymosin (RR 0.50, 95% CI: 0.26-0.97), oral transfer factor (RR 0.51, 95% CI: 0.35-0.73), BCG vaccine injection (RR 0.68, 95% CI: 0.48-0.95), *Huang qi* granules (RR 0.62, 95% CI: 0.47-0.83) and *Tiaojining* (RR 0.59, 95% CI: 0.43-0.81) were also effective in reducing the risk of infection in children with nephrotic syndrome. However mannan peptide tablet (RR 0.46, 95% CI: 0.21-1.01) and polyvalent bacterial vaccine (RR 0.24, 95% CI: 0.06-1.00) were not superior to baseline treatment in reducing the risk of infection for nephrotic children.

Honey for infected post-operative wounds [46]	Honey appeared to heal infected post-operative wounds more quickly than antiseptics and gauze	One trial (N = 50) on infected post-operative wounds. Honey healed infected post-operative wounds more quickly than antiseptic washes followed by gauze and was associated with fewer adverse events (moderate quality evidence, RR of healing: 1.69, 95% CI: 1.10-2.61).

Chinese herbal medicines for skin and soft-tissue infections [47]	No RCTs that met the inclusion criteria > No conclusion	

**Table 3:** Non-Cochrane reviews with some evidence of effectiveness of CAM treatments of infections.

Treatment and indication	Main conclusions	Study characteristics
**Respiratory tract infections (RTIs)**		

*Andrographis paniculata* for symptomatic relief of acute respiratory tract infections in adults and children [48]	*A. paniculata* appears beneficial and safe for relieving ARTI symptoms and shortening time to symptom resolution. However, these findings should be interpreted cautiously owing to poor study quality and heterogeneity. Well-designed trials evaluating the effectiveness and potential to reduce antibiotic use of *A. paniculata* are warranted	33 RCTs with a total of 7.175 patients were included. Most trials evaluating *A. paniculata* (as a monotherapy and as a herbal mixture) provided commercially but seldom reported manufacturing or quality control details. *A. paniculata* improved cough (n = 596, standardised mean difference SMD: -0.39, 95% CI: -0.67 to -0.10) and sore throat (n = 314, SMD: -1.13, 95% CI: -1.37 to -0.89) when compared with placebo. *A. paniculata* (alone or plus usual care) has a statistically significant effect in improving overall symptoms of ARTIs when compared to placebo, usual care, and other herbal therapies. Evidence also suggested that *A. paniculata* (alone or plus usual care) shortened the duration of cough, sore throat, and sick leave/time to resolution when compared with usual care. The methodological quality of included trials was overall poor.

*Pelargonium sidoides* preparation (EPS 7630) for acute bronchitis, acute rhinosinusitis and acute tonsillopharyngitis [49]	Superiority of EPS 7630 to placebo in reducing both symptom severity and time until complete recovery for all indications investigated	13 trials with a total of 3.392 participants were included, 10 of which could be entered into meta-analyses of efficacy (AB: 6/8 trials; ARS: 2/2 trials; ATP: 2/3 trials). In ARS, all trials included adults only, whereas studies in ATP had been conducted with children only. EPS 7630 was superior to placebo in reducing both symptom severity and time until complete recovery for all indications investigated. Significant advantages for the herbal drug were also observed for time until the onset of a meaningful treatment effect, global therapy outcome, and days off work, school, or kindergarten. In AB, efficacy could also be shown for both subsets defined by age.

*Pelargonium sidoides* for acute rhinosinusitis [50]	Positive evidence	Seven trials on *P. sidoides* (EPs 7630, Umckaloabo®), Myrtol (GeloMyrtol® forte), BNO 1016 (Sinupret® extract), BNO 101 (Sinupret®), *Cyclamen europaeum* (Nasodren®), and Esberitox® were included. Risk of bias was heterogeneous. EPs 7630 appeared to be useful in the treatment of ARS. Myrtol showed benefits against a placebo compound, and BNO 1016 and BNO 101 might be helpful; however, there was little evidence for the effectiveness of *Cyclamen europaeum* and Esberitox® (p-values not presented).

*Echinacea* and *Pelargonium sidoides* for treatment of RTIs in children [51]	Because of conflicting evidence in the included studies, no concrete conclusion on effects of *Echinacea* could be drawn so far. In the case of *P. sidoides*, there is moderate evidence for efficacy and safety in the treatment of RTIs in children	Eleven trials with 2.181 participants were included. No clear evidence for *Echinacea* (4 trials) or an herbal compound preparation (1 trial) in preventing RTI symptoms was found. Meta-analysis revealed evidence for efficacy (responder rates: RR: 2.56; 95% CI: 1.54 – 4.26; p < .01) and safety (patients with adverse events: RR: 1.06, 95% CI: 0.42 – 2.66; p = .9) of *P. sidoides* in treating RTI symptoms compared with placebo (6 trials).

Probiotics for prevention of upper respiratory tract infections (URTIs) in children [52]	Probiotics decrease the incidence of URTIs	23 trials with a total of 6.269 children (age: 0 -18). None of the trials showed a high risk of bias. The quality of the evidence of outcomes was moderate. Probiotic consumption significantly decreased the number of subjects having at least 1 RTI episode (17 RCTs, 4.513 children, RR: 0.89, 95% CI: 0.82–0.96, p=* *0.004). Children supplemented with probiotics had fewer numbers of days of RTIs per person compared with children who had taken a placebo (6 RCTs, 2.067 children, MD: −0.16, 95% CI: −0.29 to 0.02, p* *=* *0.03) and had fewer numbers of absence days from day care/school (8 RCTs, 1.499 children, MD: −0.94, 95% CI: −1.72 to −0.15, p* *=* *0.02). However, there was no statistically significant difference of illness episode duration between probiotic intervention group and placebo group (9 RCTs, 2.817 children, MD: −0.60, 95% CI: −1.49 to 0.30, p* *=* *0.19).

Probiotics for prevention of URTIs in immunocompetent children [53]	Modest effect both in diminishing the incidence of URTIs and the severity of the infection symptoms	14 RCTs applied to a pediatric population with high-quality methodology. At least one beneficial effect of prophylactic probiotic was observed in the majority of RCTs. The long-term administration of probiotics appeared to have a good safety profile in childhood and none of the studies reported any serious adverse events related to the probiotic strain. Probiotics in immunocompetent children have a modest effect in diminishing both the incidence of URTIs (number of subjects having at least 1 respiratory symptom episode (RR: 0.89, 95% CI: 0.82 – 0.96, p = 0.004); children supplemented with probiotics had fewer number of days of RTIs per person compared with children who had taken a placebo (weighted MD: 0.16, 95% CI: 0.29-0.02, p = 0.03)) and the severity of the infection symptoms.

*Sanren Decoction* (made of almonds, *Amomum cardamomum, barley, talc, tetrapanax papyrifera, folia bambosae, Magnolia officinalis, Pinellia ternate*) for URTIs [54]	Higher cure rate and effectiveness rate than control group High quality evidence is required	Seven studies with 571 URTI patients. The cure rate (OR = 3.51, 95% CI: 2.19-5.15, p < 0.001) and effectiveness rate (OR = 3.91, 95% CI: 2.58-5.90, p < 0.001) of *Sanren Decoction's* treatment on URTI were significantly higher than those of control group.

*Shuanghuanglian* injection for URTIs [55]	Better effect than common antibiotics on helping relieve some symptoms and decrease the course of acute upper respiratory tract infections High quality evidence is required	Eight trials with 857 participants. SHL injection showed significant effect on reducing the time to resolution of fever (3 trials, 297 patients; MD: 0.82 day, 95% CI: 0.6-1.04, p < 0.00001) and the resolution time of cough (2 trials, 209 patients; MD: 0.9 day, 95% CI: 0.58-1.23, p < 0.00001), when compared with ribavirin and/or lincomycin. SHL injections had significant effect on reducing the resolution time of sore throat (1 trial, 79 patients; MD: 1.39 day, 95% CI: 0.88-1.9) and nasal congestion and discharge (1 trial, 130 patients; MD: 0.74 day, 95% CI: 0.11-1.37) (p-values not presented).

Homeopathy for URTIs [56, 57]	Positive results	29 studies of different designs (17 RCTs) with 5.062 patients on the domain ‘Upper Respiratory Tract Infection/Allergy' (URTI/A) showed an overall positive result in favour of homeopathy. 6 out of 7 of the controlled studies demonstrated at least equivalence with conventional medical interventions and 8 out of 16 placebo controlled studies significance in favour of homeopathy. This positive trend was maintained in the evaluation of subgroups.

Individualized homeopathy for children with URTI, tonsillitis and acute sinusitis [58]	Homeopathy is a more or at least not inferior cost-effective method than placebo or conventional and antibiotic treatments	Six clinical trials (N= not presented). A significant difference in the median total symptom score in patients receiving homeopathy compared to the recipients of placebo in control groups (p = 0.026). Homeopathic strategies yielded significantly better results compared to antibiotic strategies in terms of medical efficacy (p ≤ 0.001).

Herbal medicine for cough [59]	Strong evidence for *Andrographis paniculata* and *ivy/primrose/thyme*-based preparations Moderate evidence for *Pelargonium sidoides*	34 RCTs (N = 7.083) on *P. sidoides* (11 RCTs), *Echinacea* (8 RCTs), *A. paniculata* (6 RCTs), *ivy/primrose/thyme* (4 RCTs), essential oils (4 RCTs) and *bakumondoto* (1 RCT) were included. Controls were mainly placebo. Most studies had a low risk of bias. The meta-analysis revealed strong evidence for *A. paniculata* (SMD = -1.00, 95% CI: -1.85 to -0.15; p < 0.001) and *ivy/primrose/thyme* (RR = 1.40, 95% CI: 1.23-1.60; p < 0.001) in treating cough; moderate evidence for *P. sidoides* (RR = 4.60; 95% CI: 2.89-7.31; p < 0.001), and limited evidence for *Echinacea* (SMD = -0.68; 95% CI: -1.32 to -0.04; p = 0.04).

Chinese herbal medicine for postinfectious cough [60]	Improvement of core symptoms of postinfectious cough Enhancement of quality of life	12 RCTs with moderate-to-high levels of evidence. Methodological quality was considered high in three trials, while in the other nine studies the unclear risk of bias was in the majority. Findings suggested that, compared with western conventional medicine or placebo, Chinese herbal medicine could effectively improve core symptoms of postinfectious cough, act better and have earlier antitussive effect, and enhance patients' quality of life. No serious adverse event was reported.

Chinese medicine for respiratory diseases [61]	Chinese medicine was more effective than anti-viral medicine	Six economic evaluations and cost studies were included, of which 4 studies' quality was low, 1 was high and 1 was medium. All studies adequately documented effectiveness of interventions. However, the costs of interventions were not well reported in 2 studies. 2 studies inadequately conducted sensitivity analysis and discounting. The diseases of 6 studies included bronchitis (2 studies), upper respiratory tract infection, herpangina, hand-foot-and-mouth disease and viral pneumonia. The studies results showed that cost-effectiveness of *Xiyanping* injection is poorer than *Tanreqing* injection and has more adverse reaction in 2 studies, and it is poorer than *Yanhuning* injection, but with less adverse reaction in 2 studies. *Xiyanping* injection is better than anti-viral medicine in 2 studies. 1 study indicated that *Xiyanping* is more cost-effective by atomized than intravenous drip.

**Urinary tract infections (UTIs)**		

Cranberry for UTIs [62]	Evidence supporting clinical efficacy of cranberry product UTI prophylaxis exists in the following populations: women with rUTI, women with rUTI over 49 years old, children, rUTI, post-gynecological surgery patients, patients carrying a double-J ureteral stent, high-UTI-risk long-term care facility (LTCF) patients, prostatic adenocarcinoma patients treated with radiotherapy, and renal transplant patients with rUTI. An absence of clinical efficacy for cranberry product UTI prophylaxis exists in populations of women with rUTI (other studies), elderly males and females, neuropathic bladder/spinal injury patients, pregnant women, children (other studies), radiotherapy patients, low-UTI-risk LTCF patients, and MS patients with neurogenic bladder.	22 relevant articles: three SRs, two SRs with MAs, eight RCTs, five NRSs, and four guidelines with relevant recommendations.

**Antibiotic-associated diarrhoea**		

Probiotics for antibiotic-associated diarrhoea (AAD) [63]	Reduction of AAD	A total of 82 RCTs met inclusion criteria. The majority used Lactobacillus-based interventions alone or in combination with other genera; strains were poorly documented. The pooled relative risk in a DerSimonian-Laird random-effects meta-analysis of 63 RCTs, which included 11.811 participants, indicated a statistically significant association of probiotic administration with reduction in AAD (relative risk: 0.58; 95% CI: 0.50-0.68; p < .001; I(2), 54%; [risk difference: -0.07; 95% CI, -0.10 to -0.05], [number needed to treat: 13; 95% CI: 10.3-19.1]) in trials reporting on the number of patients with AAD. This result was relatively insensitive to numerous subgroup analyses. However, there exists significant heterogeneity in pooled results and the evidence is insufficient to determine whether this association varies systematically by population, antibiotic characteristic, or probiotic preparation.

Probiotics for prevention of AAD [64]	Preventive effects on AAD in adults (18–64 years) but not the elderly (> 65 years)	30 RCTs met the predefined inclusion criteria and were included in the meta-analysis. There was considerable heterogeneity among the trials (p < .001); thus, subgroup analyses were performed. The meta-analysis resulted in a pooled relative risk (RR) of AAD of 0.69 (95% CI: 0.62-0.76) in a fixed effects model and 0.58 (95% CI: 0.48-0.71) in a random effects model, as compared with placebo. The positive association between intake of probiotic and reduced risk of AAD was observed in adults (RR: 0.47; 95% CI: 0.4-0.56). In contrast, in elderly patients, there was no positive effect (RR: 0.94; 95% CI: 0.76-1.15) of probiotic use and AAD.

## Data Availability

The datasets used and/or analyzed during the current study are available from the corresponding author on reasonable request.

## Abbreviations

95% CI: 95% confidence interval
AAD: Antibiotic-associated diarrhoea
AB: Acute bronchitis
AM: Anthroposophic medicine
AMR: Antimicrobial resistance
AOM: Acute otitis media
*A. paniculata*: * Andrographis paniculata*
ARDS: Acute respiratory distress syndrome
ARS: Acute rhinosinusitis
ARTI: Acute respiratory tract infection
ATP: Acute tonsillopharyngitis
BCG: Bacillus Calmette-Guerin
CAM: Complementary and Alternative Medicine
CD4: Cluster of differentiation 4
CHM: Chinese herbal medicine
*E. coli*: * Escherichia coli*
EBM: Evidence-based medicine
EU: European Union
GP: General practitioner
IBD: Irritable bowel disease
IBS: Irritable bowel syndrome
ICU: Intensive care unit
IFN*γ*: Interferon gamma
IM: Integrative Medicine
IVIG: Intravenous immunoglobulin
IS: Immunostimulant
LTC: Long-term care
MA: Meta-analysis
MAP: Mitogen-activated protein
MBC: Minimal bactericidal concentration
MD: Mean difference
MIC: Minimal inhibitory concentration
MPs: Medicinal products
MRSA: Multiresistant* Staphylococcus aureus*
MS: Multiple sclerosis
N: Number
NF-*κ*B: Nuclear factor kappa-light-chain-enhancer of activated B cells
NHS: National Health Service
NRS: Nonrandomized controlled study
OR: Odds ratio
PCA: Protocatechuic acid
*P. sidoides*: * Pelargonium sidoides*
RCT: Randomized controlled trial
RR: Relative risk
RTI: Respiratory tract infection
rUTI: Recurrent urinary tract infection
*S. aureus*: * Staphylococcus aureus*
SIBO: Small Intestinal Bacterial Overgrowth
SMD: Standardized mean difference
*S. typhimurium*: * Salmonella typhimurium*
TNF*α*: Tumor necrosis factor alpha
TTO: Tea tree oil
UK: United Kingdom
URTI: Upper respiratory tract infection
UTI: Urinary tract infection
USA: United States of America
WHO: World Health Organization.
