# Corrigendum to “The Contribution of Complementary and Alternative Medicine to Reduce Antibiotic Use: A Narrative Review of Health Concepts, Prevention, and Treatment Strategies”

**DOI:** 10.1155/2020/7089287

**Published:** 2020-09-18

**Authors:** Erik W. Baars, Eefje Belt-van Zoen, Thomas Breitkreuz, David Martin, Harald Matthes, Tido von Schoen-Angerer, Georg Soldner, Jan Vagedes, Herman van Wietmarschen, Olga Patijn, Merlin Willcox, Paschen von Flotow, Michael Teut, Klaus von Ammon, Madan Thangavelu, Ursula Wolf, Josef Hummelsberger, Ton Nicolai, Philippe Hartemann, Henrik Szőke, Michael McIntyre, Esther T. van der Werf, Roman Huber

**Affiliations:** ^1^Louis Bolk Institute, Kosterijland 3-5, 3981 AJ Bunnik, Netherlands; ^2^University of Applied Sciences Leiden, Faculty of Healthcare, Zernikedreef 11, 2333 CK Leiden, Netherlands; ^3^Filderklinik, Im Haberschlai 7, 70794 Filderstadt, Germany; ^4^University of Witten/Herdecke, Alfred-Herrhausen-Strabe 50, 58448 Witten, Germany; ^5^Charité Universitätsmedizin Berlin, Institute for Social Medicine, Epidemiology and Health Economics, Luisenstr 57, 10117 Berlin, Germany; ^6^Department of Pediatrics, Fribourg Hospital HFR, Fribourg, Switzerland; ^7^Medical Section of the Goetheanum, Rüttiweg 45, 4143 Dornach, Switzerland; ^8^ARCIM Institute, Im Haberschlai 7, 70794 Filderstadt, Germany; ^9^University of Southampton, University Road, Southampton SO17 1BJ, UK; ^10^Sustainable Business Institute, Zehnthofstr 1, 65375 Oestrich-Winkel, Germany; ^11^University of Bern, Institute of Complementary and Integrative Medicine, Freiburgstrasse 46, 3010 Bern, Switzerland; ^12^European Ayurveda Association e.V., In den Forstwiesen 27, D-56745 Bell, Germany; ^13^Technical University Munich, Georg-Brauchle-Ring 62, 80807 Munich, Germany; ^14^Eurocam, Rue du Trône 194, 1050 Brussels, Belgium; ^15^University of Lorraine, School of Medicine, 7 Avenue de la Forêt de Haye, 54500 Vandoeuvre-Nancy, France; ^16^University of Pécs, 7622 Pécs, Vasvári Pál Str. 4, Hungary; ^17^Midsummer Clinic, Church Westcote, Chipping Norton, Oxon Ox7 6SF, UK; ^18^Taylor's University, School of Medicine, 1, Jalan Taylor's, 47500 Subang Jaya, Selangor D.E., Malaysia; ^19^University of Bristol, Bristol Medical School, Canynge Hall, 39 Whatley Road, Bristol BS8 2PS, UK; ^20^University of Freiburg, Faculty of Medicine, Breisacher Str. 115b, 79106 Freiburg, Germany

In the article titled “The Contribution of Complementary and Alternative Medicine to Reduce Antibiotic Use: A Narrative Review of Health Concepts, Prevention, and Treatment Strategies” [1], affiliation 11 was incomplete. Therefore, it is updated from “University of Bern, Freiburgstrasse 46, 3010 Bern, Switzerland,” to “University of Bern, Institute of Complementary and Integrative Medicine, Freiburgstrasse 46, 3010 Bern.” This affects the affiliation details for authors Ursula Wolf and Klaus von Ammon. The corrected affiliation is updated in the abovementioned author information.
